# Supplementary material for: Repetitive Low-Intensity Vestibular Noise Stimulation Partly Reverses Behavioral and Brain Activity Changes following Bilateral Vestibular Loss in Rats
Source: Biomolecules. 2023 Oct 26;13(11):1580. doi: 10.3390/biom13111580 (PMC10669117; doi:10.3390/biom13111580)
Supplement: Supplementary file 1 [file biomolecules-13-01580-s001.zip › biomolecules-2611963-supplementary.pdf]

	TIMEPOINT	baseline	day 1		day 3		day 7		day 15		day 30		day 60		STATISTIC<br>factor timepoint
	GROUP		sham	nGVS	sham	nGVS	sham	nGVS	sham	nGVS	sham	nGVS	sham	nGVS	factor group
HEMISPHERIC SENSOR NETWORKS	olfactory	104.3 (6.4)	100.5 (4.8)	103.0 (4.0)	98.8 (4.2)	102.4 (5.0)	101.2 (6.6)	101.2 (3.6)	99.8 (10.8)	99.3 (5.7)	102.4 (2.7)	98.2 (3.3)	100.6 (3.6)	100.4 (5.1)	p = 0.664, η_p^2 = 0.025<br>p = 0.721, η_p^2 = 0.005
	auditory	112.1 (2.6)	115.5 (3.8)	112.4 (3.7)	111.8 (5.7)	110.3 (4.5)	112.3 (7.6)	105.9 (6.6)	109.8 (5.5)	108.2 (3.3)	110.3 (3.7)	108.9 (4.5)	106.6 (4.8)	108.0 (3.9)	**p = 0.004**, η_p^2 = 0.130<br>p = 0.211, η_p^2 = 0.062
	insular c.	113.0 (5.2)	118.3 (3.3)	121.2 (4.9)	119.1 (4.4)	120.4 (5.6)	119.9 (4.9)	122.6 (4.3)	122.9 (4.4)	116.1 (5.3)	119.6 (3.8)	119.4 (6.3)	116.3 (8.5)	120.3 (5.1)	**p < 0.001**, η_p^2 = 0.230<br>p = 0.395, η_p^2 = 0.029
	somato-sensory c.	112.1 (3.3)	121.0 (4.4)	123.6 (4.8)	121.2 (2.5)	124.9 (4.1)	124.8 (6.4)	128.9 (2.0)	123.8 (3.7)	120.5 (5.2)	118.7 (3.1)	126.2 (4.6)	113.2 (8.8)	118.2 (3.9)	**p < 0.001**, η_p^2 = 0.241<br>**p = 0.004**, η_p^2 = 0.286
	parietal c.	109.5 (3.0)	118.8 (3.3)	117.0 (4.5)	116.2 (4.3)	117.9 (3.9)	119.5 (7.2)	112.7 (5.0)	116.0 (9.4)	113.0 (5.0)	114.8 (2.0)	115.1 (4.2)	106.9 (13.4)	109.0 (6.7)	**p < 0.001**, η_p^2 = 0.188<br>p = 0.274, η_p^2 = 0.048
	visual c.	109.6 (3.2)	117.0 (4.1)	113.6 (4.8)	113.8 (4.5)	112.5 (5.3)	112.5 (7.4)	107.0 (6.5)	109.5 (12.6)	111.4 (4.0)	113.2 (4.8)	109.6 (5.5)	107.7 (10.7)	109.5 (6.2)	**p = 0.041**, η_p^2 = 0.088<br>p = 0.527, η_p^2 = 0.016
	lateral thalamus	119.4 (6.1)	124.4 (3.8)	127.9 (5.7)	118.5 (7.8)	127.0 (5.3)	128.1 (7.5)	127.2 (8.1)	126.6 (4.9)	123.8 (7.5)	131.0 (8.1)	128.1 (5.6)	129.3 (7.2)	127.2 (9.8)	**p = 0.001**, η_p^2 = 0.087<br>p = 0.305, η_p^2 = 0.042
HEMISPHERIC MOTOR NETWORKS	orbito-frontal c.	120.7 (7.8)	125.9 (4.0)	127.3 (7.0)	123.5 (3.4)	127.3 (7.0)	119.5 (2.6)	124.9 (7.2)	123.4 (6.7)	124.8 (6.5)	124.2 (3.2)	120.4 (4.3)	119.4 (11.1)	125.0 (7.0)	**p = 0.004**, η_p^2 = 0.080<br>p = 0.121, η_p^2 = 0.093
	medial pre-frontal c.	127.5 (5.7)	137.2 (7.7)	138.2 (5.0)	135.2 (6.2)	136.4 (5.8)	131.3 (5.4)	133.3 (5.2)	132.6 (6.6)	134.6 (4.6)	132.3 (7.6)	128.8 (2.9)	124.2 (5.1)	132.5 (6.6)	**p < 0.001**, η_p^2 = 0.157<br>**p = 0.015**, η_p^2 = 0.214
	frontal association c.	109.4 (7.1)	108.5 (7.2)	109.1 (4.3)	104.5 (7.6)	111.2 (7.0)	100.6 (5.3)	110.7 (8.0)	108.3 (7.0)	113.7 (6.8)	113.4 (4.9)	111.5 (7.4)	101.7 (17.2)	112.2 (11.2)	p = 0.069, η_p^2 = 0.078<br>**p = 0.005**, η_p^2 = 0.273
	motor c.	117.1 (3.2)	120.9 (2.8)	124.2 (4.2)	121.3 (3.9)	126.2 (3.2)	121.0 (3.7)	128.7 (4.6)	123.0 (4.3)	124.1 (4.5)	122.9 (3.4)	127.3 (5.6)	110.2 (14.8)	122.9 (4.2)	**p < 0.001**, η_p^2 = 0.158<br>**p < 0.001**, η_p^2 = 0.504
	striatum	121.2 (4.3)	128.4 (4.1)	134.3 (5.6)	129.0 (3.3)	133.2 (4.6)	132.6 (7.7)	138.7 (4.6)	127.8 (4.9)	126.5 (3.9)	125.1 (5.0)	134.4 (6.0)	123.0 (4.0)	131.2 (5.2)	**p < 0.001**, η_p^2 = 0.171<br>**p < 0.001**, η_p^2 = 0.462
LIMBIC NETWORKS	accumbens	116.5 (6.8)	117.6 (3.6)	124.4 (5.3)	114.1 (6.5)	122.6 (7.8)	119.4 (9.6)	121.3 (5.1)	114.1 (6.5)	122.6 (7.8)	119.4 (9.6)	121.3 (5.1)	114.0 (6.5)	114.6 (4.5)	**p = 0.001**, η_p^2 = 0.215<br>**p = 0.015**, η_p^2 = 0.116
	amygdala	97.8 (2.4)	97.8 (3.4)	100.2 (4.4)	98.9 (2.3)	100.8 (4.7)	100.4 (4.0)	100.6 (6.3)	96.5 (3.5)	98.3 (4.0)	98.1 (2.6)	99.4 (4.1)	97.1 (4.5)	102.4 (1.6)	p = 0.139, η_p^2 = 0.029<br>p = 0.171, η_p^2 = 0.074
	entorhinal c.	103.9 (2.7)	107.6 (3.7)	108.0 (2.9)	107.4 (5.2)	108.3 (3.9)	110.5 (3.5)	108.5 (3.9)	107.8 (3.7)	105.5 (3.6)	107.5 (4.3)	108.4 (5.6)	104.6 (3.5)	108.6 (6.3)	**p = 0.044**, η_p^2 = 0.089<br>p = 0.481, η_p^2 = 0.020
	ad. hippocampus	104.5 (3.3)	109.2 (2.4)	107.0 (3.8)	109.0 (2.7)	108.6 (3.2)	106.4 (2.7)	106.3 (4.0)	107.2 (4.5)	106.6 (3.9)	110.3 (2.5)	105.2 (4.0)	108.9 (2.8)	108.6 (3.7)	p = 0.059, η_p^2 = 0.067<br>p = 0.806, η_p^2 = 0.002
	p. hippocampus	99.7 (8.1)	99.7 (3.8)	95.7 (4.8)	98.4 (6.4)	99.4 (5.4)	95.4 (5.7)	90.0 (5.5)	97.4 (5.4)	96.6 (4.8)	101.5 (2.7)	94.6 (6.1)	99.3 (5.4)	99.3 (7.1)	**p = 0.008**, η_p^2 = 0.117<br>p = 0.177, η_p^2 = 0.072
	cingulate	125.8 (4.4)	131.4 (8.2)	136.3 (6.6)	135.1 (4.8)	138.0 (3.2)	132.7 (5.5)	140.6 (5.8)	136.4 (5.1)	135.7 (4.4)	133.9 (8.4)	139.3 (7.9)	124.3 (8.4)	134.8 (4.9)	**p < 0.001**, η_p^2 = 0.137<br>**p < 0.001**, η_p^2 = 0.506
	retrosplenial c.	109.1 (6.6)	109.5 (4.6)	108.8 (3.8)	111.0 (4.8)	112.4 (3.4)	112.4 (5.8)	111.8 (6.6)	110.7 (9.2)	112.3 (4.9)	114.0 (4.5)	112.6 (4.4)	109.6 (5.9)	115.1 (5.4)	p = 0.343, η_p^2 = 0.038<br>**p = 0.021**, η_p^2 = 0.195
BRAINSTEM-CEREBELLAR NETWORKS	cerebellar g.m.	94.8 (1.9)	93.6 (5.3)	92.7 (4.6)	90.3 (2.0)	89.8 (5.2)	91.3 (5.3)	90.0 (4.0)	92.0 (5.5)	91.8 (5.0)	95.8 (2.7)	89.8 (7.4)	99.7 (5.9)	93.1 (5.9)	**p = 0.011**, η_p^2 = 0.111<br>**p < 0.001**, η_p^2 = 0.428
	cerebellar w.m.	107.5 (4.9)	110.2 (6.0)	106.9 (6.2)	103.6 (3.6)	103.0 (6.3)	106.3 (7.9)	104.8 (3.2)	105.7 (6.3)	105.0 (5.5)	107.8 (3.7)	102.7 (7.8)	111.8 (4.9)	106.0 (6.9)	p = 0.053, η_p^2 = 0.083<br>**p = 0.004**, η_p^2 = 0.293
	v. tegmental area	106.3 (5.7)	108.3 (4.5)	107.4 (7.5)	113.4 (6.3)	108.1 (6.1)	105.5 (4.4)	106.1 (7.6)	109.3 (4.0)	108.8 (4.1)	107.5 (3.0)	105.8 (7.7)	110.1 (3.6)	111.1 (7.1)	p = 0.259, η_p^2 = 0.100<br>p = 0.803, η_p^2 = 0.003
	colliculus superior	119.3 (5.7)	112.8 (4.8)	112.2 (5.3)	114.7 (2.1)	110.7 (5.8)	110.4 (6.1)	110.5 (3.7)	111.7 (6.5)	115.2 (2.3)	113.3 (2.6)	109.6 (6.6)	114.4 (5.9)	114.3 (5.0)	**p = 0.005**, η_p^2 = 0.168<br>p = 0.277, η_p^2 = 0.047
	colliculus inferior	127.8 (6.6)	108.5 (4.5)	104.5 (4.3)	108.7 (2.4)	105.3 (6.8)	104.5 (4.6)	103.7 (4.4)	105.8(6.5)	106.5 (4.5)	106.7 (2.7)	102.4 (5.9)	109.7 (5.2)	110.2 (4.0)	**p < 0.001**, η_p^2 = 0.134<br>**p = 0.006**, η_p^2 = 0.265
	pons	99.3 (4.1)	95.2 (4.6)	88.6 (4.9)	95.4 (5.0)	91.1 (6.0)	92.5 (5.9)	92.4 (4.4)	96.9 (6.1)	91.8 (3.7)	92.9 (3.1)	87.1 (4.5)	93.5 (6.2)	91.0 (4.6)	**p = 0.002**, η_p^2 = 0.145<br>**p = 0.043**, η_p^2 = 0.299
	vestibular nucleus	116.7 (4.4)	105.1 (6.2)	98.6 (6.2)	105.0 (5.8)	101.5 (7.6)	100.9 (10.9)	100.4 (5.8)	105.3 (5.2)	103.0 (4.5)	102.4 (4.1)	96.4 (6.3)	105.7 (8.8)	99.5 (5.4)	**p < 0.001**, η_p^2 = 0.152<br>**p = 0.009**, η_p^2 = 0.246

Suppl. Table S1. Descriptive statistics (mean ± SD) and statistical comparisons of changes in mean normalized levels of brain activity in 26 selected brain regions measured by [^{18}F]-FDG-PET.
